# Machine Learning–Based Prediction Models for Different Clinical Risks in Different Hospitals: Evaluation of Live Performance

**DOI:** 10.2196/34295

**Published:** 2022-06-07

**Authors:** Hong Sun, Kristof Depraetere, Laurent Meesseman, Patricia Cabanillas Silva, Ralph Szymanowsky, Janis Fliegenschmidt, Nikolai Hulde, Vera von Dossow, Martijn Vanbiervliet, Jos De Baerdemaeker, Diana M Roccaro-Waldmeyer, Jörg Stieg, Manuel Domínguez Hidalgo, Fried-Michael Dahlweid

**Affiliations:** 1 Dedalus Healthcare Antwerp Belgium; 2 Institute of Anesthesiology and Pain Therapy Heart and Diabetes Centre North Rhine-Westphalia University Hospital of Ruhr-University Bochum Bad Oeynhausen Germany

**Keywords:** machine learning, clinical risk prediction, prediction, model, model evaluation, scalability, risk, live clinical workflow, delirium, sepsis, acute kidney injury, kidney, EHR, electronic health record, workflow, algorithm

## Abstract

**Background:**

Machine learning algorithms are currently used in a wide array of clinical domains to produce models that can predict clinical risk events. Most models are developed and evaluated with retrospective data, very few are evaluated in a clinical workflow, and even fewer report performances in different hospitals. In this study, we provide detailed evaluations of clinical risk prediction models in live clinical workflows for three different use cases in three different hospitals.

**Objective:**

The main objective of this study was to evaluate clinical risk prediction models in live clinical workflows and compare their performance in these setting with their performance when using retrospective data. We also aimed at generalizing the results by applying our investigation to three different use cases in three different hospitals.

**Methods:**

We trained clinical risk prediction models for three use cases (ie, delirium, sepsis, and acute kidney injury) in three different hospitals with retrospective data. We used machine learning and, specifically, deep learning to train models that were based on the Transformer model. The models were trained using a calibration tool that is common for all hospitals and use cases. The models had a common design but were calibrated using each hospital’s specific data. The models were deployed in these three hospitals and used in daily clinical practice. The predictions made by these models were logged and correlated with the diagnosis at discharge. We compared their performance with evaluations on retrospective data and conducted cross-hospital evaluations.

**Results:**

The performance of the prediction models with data from live clinical workflows was similar to the performance with retrospective data. The average value of the area under the receiver operating characteristic curve (AUROC) decreased slightly by 0.6 percentage points (from 94.8% to 94.2% at discharge). The cross-hospital evaluations exhibited severely reduced performance: the average AUROC decreased by 8 percentage points (from 94.2% to 86.3% at discharge), which indicates the importance of model calibration with data from the deployment hospital.

**Conclusions:**

Calibrating the prediction model with data from different deployment hospitals led to good performance in live settings. The performance degradation in the cross-hospital evaluation identified limitations in developing a generic model for different hospitals. Designing a generic process for model development to generate specialized prediction models for each hospital guarantees model performance in different hospitals.

## Introduction

Machine learning algorithms for clinical risk predictions are widely used in health care research and applications [1-5]. While much work has been done on developing distinct clinical risk prediction models, the scalability of the prediction models has been much less explored (ie, the extensibility of the risk prediction model for multiple diseases over different hospitals) [6].

Rajkomar et al [6] designed a single data structure based on the FHIR (Fast Healthcare Interoperability Resources) standard [7] and developed different clinical scenarios over two hospitals with this common data structure. That was the first study that reported the performance of prediction models of multiple use cases in different hospitals. Churpek et al [8] aggregated the electronic health record (EHR) from five hospitals to train a single model to make predictions on cardiac arrest, intensive care unit (ICU) transfers, or death on wards. The performance of the model outperforms the existing Modified Early Warning Score. The limitation is that both studies [6,8] were validated with retrospective data and have not yet been used in a live clinical workflow.

In our previous publication [9], we discussed the scalability issue in clinical risk prediction model development; we also presented a scalable approach for prediction model development that is applied to delirium, sepsis, and acute kidney injury (AKI) covering four different hospitals. However, these prediction models were only evaluated on retrospective data.

Evaluating the prediction models in live clinical settings is crucial because factors such as interoperability across different platforms or different prevalence can affect the performance of an artificial intelligence (AI) algorithm [10,11]. However, very few prediction models have been evaluated in a live clinical workflow. For example, several delirium prediction models that have been reported in recent years [9,12,13] have all been evaluated on retrospective data. Jauk et al [14] claimed their findings to be the only delirium prediction model that has been evaluated in a live clinical workflow. In their study, 5530 predictions were analyzed, and 119 predictions were compared with ratings of clinical experts during a period of 7 months. The limitation of Jauk et al [14] is that their model only evaluated in a single hospital. When a prediction model is evaluated in different hospitals, the performance may degrade due to the difference in EHRs and workflows between the training data and the target hospital. Wong et al [15] reported large performance degradation on sepsis prediction when a sepsis prediction model was applied in a different hospital.

Wu et al [16] considered it important to evaluate AI-based medical devices over different sites with live clinical settings to address the shortcomings, such as overfitting to training data and bias against underrepresented subgroups, among others. They investigated 130 US Food and Drug Administration–approved AI devices: 126 evaluations were performed as retrospective studies and 93 devices did not have multiple site evaluations.

In this paper, we evaluated clinical risk prediction models (ie, delirium, sepsis, and AKI) in live clinical workflows in three different hospitals in Germany. We compared the performance of the models with their performance on retrospective data from our previous work. By logging prediction requests in the production EHR system, we ran cross-hospital evaluations mimicking the performance of a prediction model in live clinical workflows of different target hospitals. Domain experts executed preliminary evaluations on clinical soundness and usefulness of the predictions by following the use of the prediction service in their daily practice.

To the best of our knowledge, we are the first to report the evaluation of machine learning–based clinical risk prediction models in the settings of production EHR systems, which focuses on evaluating several diseases in different hospitals at the same time. In addition, in the cross-hospital evaluation, we simulated the performance of a prediction model in live clinical workflows of different target hospitals.

## Methods

### Overview

We used a scalable approach, implemented in a calibration tool, to generate clinical risk prediction models for different use cases in three different German hospitals based on retrospective EHR data: Marienhospital Stuttgart (from 2004 to 2020), Herz- und Diabeteszentrum Nordrhein-Westfalen Bad Oeynhausen (from 2009 to 2020), and Medius Klinik Nürtingen (from 2009 to 2020). The evaluation in live systems was performed in the first half of 2021; details of the evaluation period are provided in Table S1 in Multimedia Appendix 1. The characteristics of the training set are provided in Table S2 in Multimedia Appendix 1, and the characteristics of the evaluation samples, in live systems, are provided in Table S3 in Multimedia Appendix 1. We refer to these three hospitals as hospital M, hospital H, and hospital N, respectively. The calibration process that generates prediction models is described in our previous work [9]. Using the calibration tool, models were trained independently on data from each hospital and deployed in the prediction service of the same hospital. Requests for predictions were generated from the EHR system in the FHIR [7] “RiskAssessment” format and were sent to the prediction service. The prediction service parsed each prediction request into an observation, which was used to generate a prediction. The predictions were returned and displayed in the EHR system. The observation, together with the corresponding risk score produced by the prediction model, were stored for further evaluation of model performance.

### Model Development and Deployment

**Figure 1:**
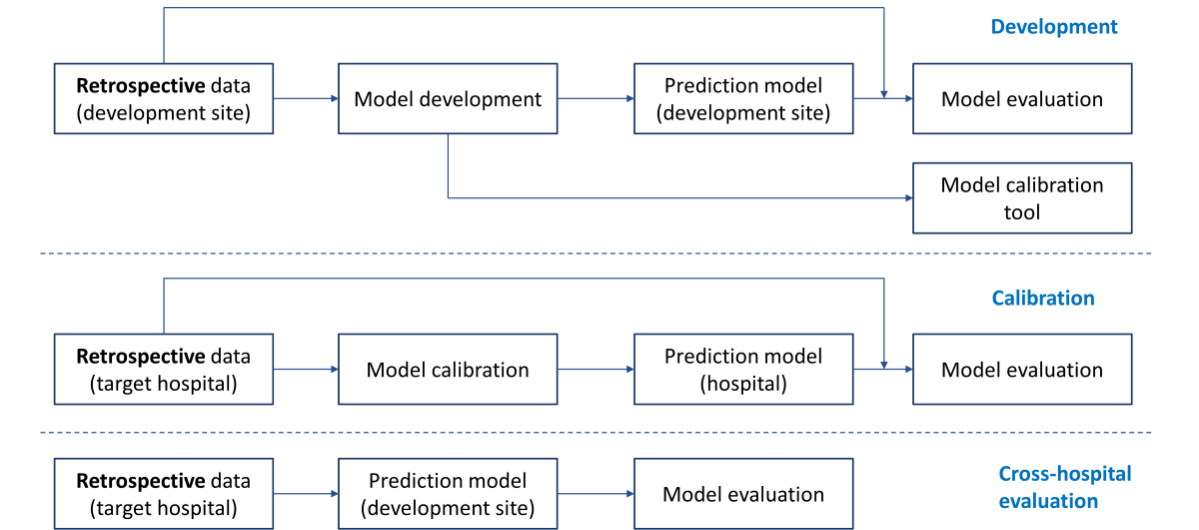
Model development and evaluation with retrospective data.

Figure 1 shows the process of model development and evaluation with retrospective data. The process to design the prediction model, prepare data, and train models was defined following experiments performed on a development data set. The resulting dedicated process and prediction model design was implemented in an automated pipeline, named the calibration tool. The calibration tool provided a user-friendly approach to install, configure, and run the process of data preparation, model training, and evaluation on a customer-specific system. A command-line interface enabled service engineers to install the required software, files, and pretrained natural language processing (NLP) models and to execute the training and evaluation of the hospital-specific prediction models.

**Figure 2:**
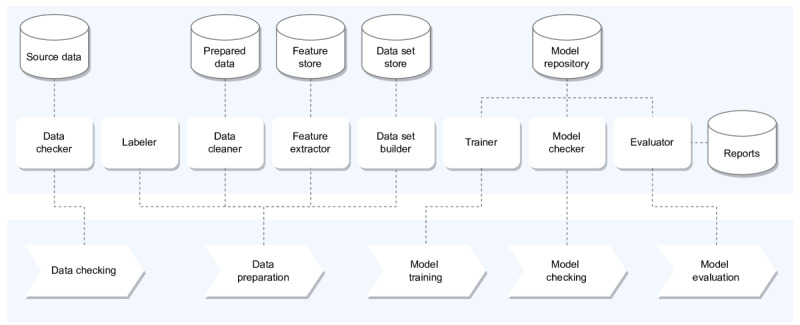
Calibration tool.

Figure 2 shows the components and interactions of the calibration tool. The lower pane defines a fixed sequence of tasks to perform in order to calibrate models for the supported use cases with data from a target hospital. The upper pane contains a set of components that execute these tasks.

We then ran the calibration tool independently in each hospital to generate clinical risk prediction models for each hospital. The models were trained based on the retrospective data that were generated as part of the clinical workflow of each target hospital. We thereby ensured that the model fit the clinical practice of the hospital where the model was to be deployed. The data checking process guaranteed that the source data were represented in the expected format. The data preparation process prepared the training and testing data. The labels of each use case were assigned by the labeler component based on the diagnosis codes assigned to each hospitalized patient at discharge. A common set of features was prepared and used by the different use cases, which included structured data, such as lab results and history of diagnosis, as well as clinical entities extracted from free-text clinical notes. Both a text search and a BERT (bidirectional encoder representations from transformers) [17] named entity recognition model were used in preparing the NLP features. The following inclusion criteria were applied during data preparation: age and gender had to be known, patients had to be 18 years or older, only inpatients could be included, and length of stay had to be limited to 90 days.

Prediction models were trained using a common model training strategy: we used the Transformer model [18] to train a binary classification model for clinical risk prediction. We concatenated the features as inputs and used the labels as targets for the model training process. The models were trained with patient data that were collected at the time of discharge with leaking features removed. In order to cope with the situation where the model was requested to make predictions when less information was available, we applied data augmentation in training sample preparation: we generated partial records in combination with the complete records to enhance the robustness of the clinical risk prediction model. More details can be found in our previous work [9]. The generated models were first examined with a model checking process, where a set of minimum requirements were assessed as unit tests. Models that passed the checks were further evaluated on their performance using common metrics, such as the area under the receiver operating characteristic curve (AUROC), sensitivity, and specificity, among others. Acceptance criteria were checked during the model evaluation process. The acceptance criteria differed among use cases and were checked for each department. Models that met the criteria could be activated in the corresponding departments to trigger alerts in the production EHR system. The acceptance criteria were complex and are explained in detail in Table S4 in Multimedia Appendix 1.

Risk prediction models evaluated in this paper were generated by our calibration tool in the three aforementioned German hospitals. The details of feature engineering and model training were presented in a former publication [9]; examples of model input features are provided in Table S5 in Multimedia Appendix 1. Preliminary cross-hospital evaluation was performed with retrospective data in our previous study and performance degradation was observed [9].

### Model Evaluation With Live Data From the Clinical Workflow

**Figure 3:**
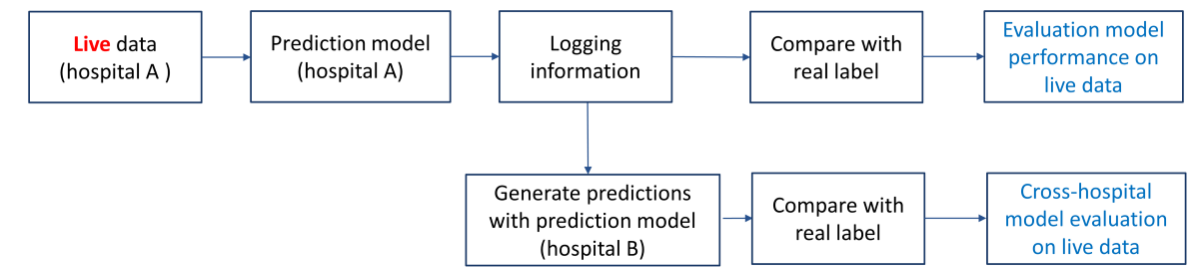
Model evaluation with live data from the clinical workflow. Hospital A refers to the hospital where risk predictions in a live electronic health record system are made. Hospital B refers to a different hospital where a different risk prediction model is trained.

Figure 3 shows the process of model evaluation with live data from the clinical workflow. Prediction services were triggered following clinical events in the EHR system (eg, when new lab results for a patient were added to the system). The EHR system sent the relevant patient record to the prediction service, where the hospitals’ specialized prediction models for three different use cases, trained on the hospital data, were deployed. For each use case, the prediction model predicted the risk of developing the related disease and returned the risk score in response. Based on the defined thresholds, alerts were created in the EHR system for those that were predicted as high risk. For each prediction made by the prediction service, the corresponding request and response were stored by a logging service. By comparing the predictions made by the prediction service and the corresponding real labels, we evaluated the model performance in a live clinical workflow. Moreover, the prediction requests stored by the logging service could be used to generate predictions with a different model to simulate its performance in a live clinical workflow. This alternate model could be a model that is trained in the same hospital with a different training strategy, as well as a model that is trained on the data from a different hospital. For example, in Figure 3, the logging information stored in hospital A (ie, the hospital where risk predictions in a live EHR system are made) can be used to generate predictions with a model trained at hospital B (ie, a different hospital where a different risk prediction model is trained). By comparing those predictions with the real labels, it is possible to estimate the performance of the model of hospital B in the live clinical workflow of hospital A.

To support the evaluation presented in this paper, the JSON file logging driver (ie, the default Docker logging service) [19] was used to log the request and response of prediction services to separate JSON log files. Each prediction request log entry contained the date and time and the input features for the prediction. Each prediction response log entry contained the used input features and risk score for the prediction. An excerpt of a sample log of prediction requests is enclosed (Table S5 in Multimedia Appendix 1). In the production EHR system, the prediction service can process a patient’s records and instantly make a corresponding prediction or explanation. Prediction models for delirium, sepsis, and AKI were installed in three hospitals. Prediction requests and responses were logged in these three hospitals as input for the evaluations. The response time for predictions was evaluated and provided (Figure S1 in Multimedia Appendix 1).

### Ethical Considerations

Our study to assess model performance involved the analysis of unidentifiable patients, and data use was granted to us by the pilot hospitals—hospital H, hospital M, and hospital N—for this purpose, after appropriate review. Therefore, no ethics approval by the Institutional Review Board was required. The cohort study at hospital H was approved by the Ethics Committee of the Medical Faculty of the Ruhr-Universität Bochum (file No. Az.2021-861).

## Results

### Evaluation of Model Performance in Live EHR Systems

The models made predictions at different stages during a patient stay with live data; however, the performance of the clinical risk prediction models within a live clinical workflow was evaluated at the end of the day of admission, as well as on the day of discharge. The reason we checked the performance of our prediction model at these two stages was to evaluate their performance when there were limited data compared to when sufficient data were available. Leaking information, such as strong diagnostic data or textual references to the diseases to be predicted, was excluded, following the settings we applied when we evaluated the model performance on retrospective data in our previous study [9]. Taking these same strategies allowed a fair comparison between the performance achieved on live data with that obtained on retrospective data. The model performance was evaluated by the AUROC. We choose to evaluate using the AUROC because the sensitivity, specificity, and precision were dependent on the threshold (ie, defined by the point chosen on the receiver operating characteristic curve). The threshold is used by the hospitals to trigger an alert and may differ among hospitals because some hospitals favor sensitivity over specificity or vice versa. Using the AUROC allowed us to compare the outcome of three use cases at three different hospitals independently from this threshold. Sensitivity, specificity, and precision were used to decide on the threshold and are provided with the explanation of the model acceptance criteria (Table S4 in Multimedia Appendix 1).

**Figure 4:**
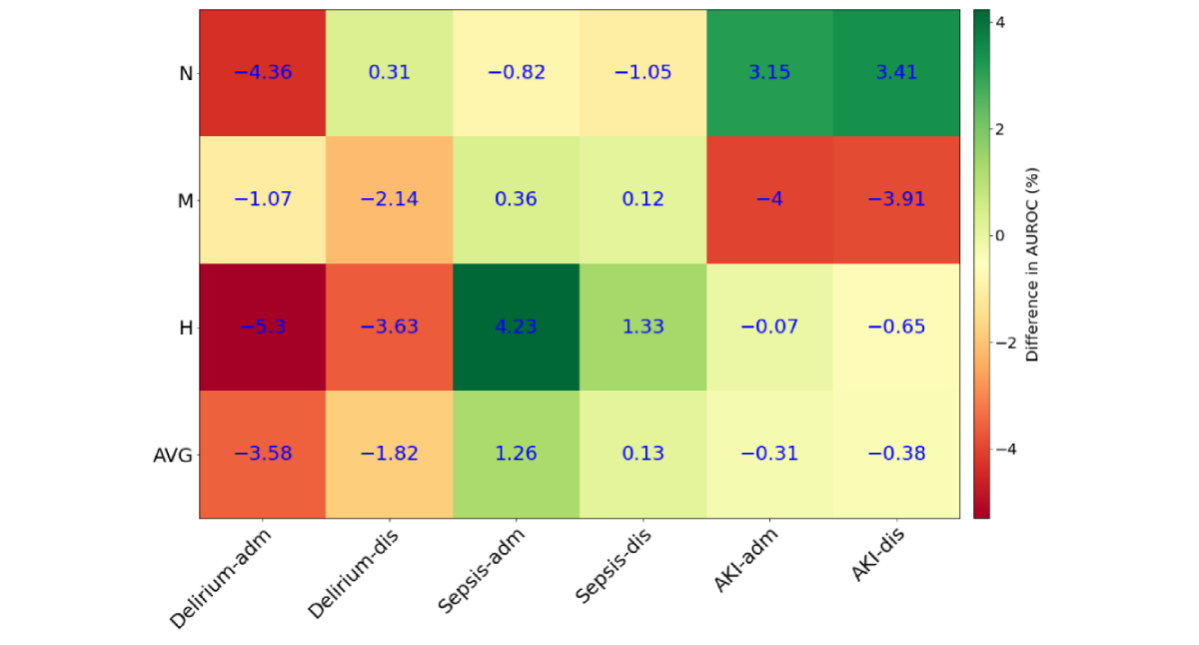
Model performance: live data vs retrospective data. The table was generated using the AUROC values for the live and retrospective data (Table S6 in Multimedia Appendix 1). adm: admission; AKI: acute kidney injury; AVG: average; AUROC: area under the receiver operating characteristic curve; dis: discharge; H: Herz- und Diabeteszentrum Nordrhein-Westfalen Bad Oeynhausen; M: Marienhospital Stuttgart; N: Medius Klinik Nürtingen.

Figure 4 evaluates the model performance as assessed by the AUROC on the live data versus the retrospective data (Table S6 in Multimedia Appendix 1). Each row in the table indicates the hospital in which the evaluation was performed. Each column indicates the use case and the point in time of the model evaluation (ie, either at the end of the admission day or at discharge). Positive values (ie, shades of green) indicate that the respective model performed better when evaluated on live data as compared to retrospective data, whereas negative values (ie, shades of red) indicate that models performed worse when evaluated on live data as compared to retrospective data. For example, the delirium model AUROC, evaluated at the end of the day of admission at hospital N, was 4.36 percentage points lower when the model was performed on the live data (AUROC=80.9%) as compared to retrospective data (AUROC=85.26%). On average, our delirium prediction model had a lower AUROC when evaluated on live data as compared to retrospective data. In contrast, our sepsis prediction model performed better on live data as compared to retrospective data, whereas the AKI prediction model performed equally well on both. At the hospital level, evaluation on live data led to higher model performance in hospital N (+0.1 percentage points) but to lower performance in hospitals M and H (–1.8 and –0.7 percentage points, respectively). When averaged across all three use cases and all three hospitals, the performance of our prediction models declined slightly when evaluated on live data (AUROC values: 83.1% at admission, 94.2% at discharge, and 88.6% on average) as compared to retrospective data (AUROC values: 83.0% at admission, 94.8% at discharge, and 89.4% on average).

### Cross-Hospital Evaluation

Cross-hospital evaluation was performed by extracting the observations from the prediction request in one hospital and generating predictions using a model trained on data from a different hospital. We evaluated our models in a live clinical workflow based on the logging information stored in the prediction service. The prediction requests made at different stages of a medical stay were used to generate corresponding predictions by prediction models of other hospitals. By using the prediction models of other hospitals, we simulated the performance of these models in a live clinical workflow, without the model being installed on-site.

**Figure 5:**
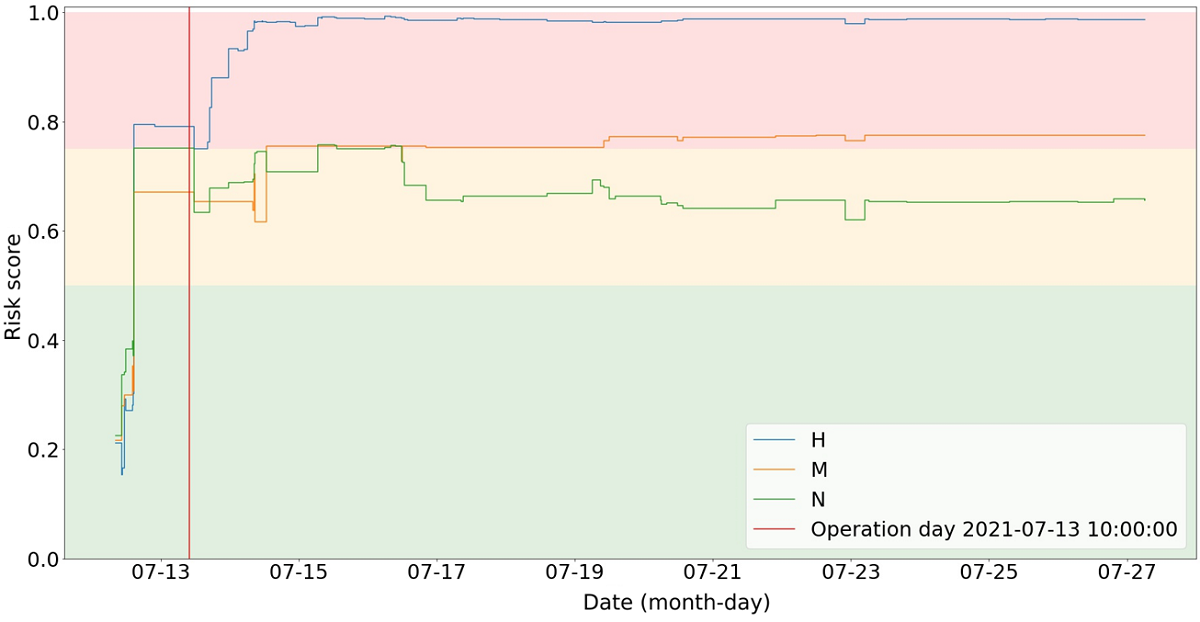
Delirium risk prediction of a sample patient during his medical stay based on data from the live electronic health record system. H: Herz- und Diabeteszentrum Nordrhein-Westfalen Bad Oeynhausen; M: Marienhospital Stuttgart; N: Medius Klinik Nürtingen.

Figure 5 shows an example of simulating the performance of models trained on data from hospitals M and N, but applied to live data of the medical stay of a sample patient in hospital H. The red vertical line indicates the point in time of the patient’s surgery. The three colored lines reflect the simulated model prediction over the course of the patient’s medical stay in hospital H, using models trained separately on data from hospital H, M, and N.

In the presented case, postoperative delirium was confirmed by an independent evaluation—the Confusion Assessment Method for the ICU (CAM-ICU) [20]—on the first postoperative day. The CAM-ICU evaluation was not included as a feature of our training model. Of the three models, the one trained at hospital H predicted the risk of delirium before surgery and identified an increased risk after surgery. The risk after surgery increased gradually when lab results and clinical entities were added. The models trained at the other hospitals both predicted the risk of delirium before surgery, but both failed to properly identify the severity of the risk after surgery.

**Figure 6:**
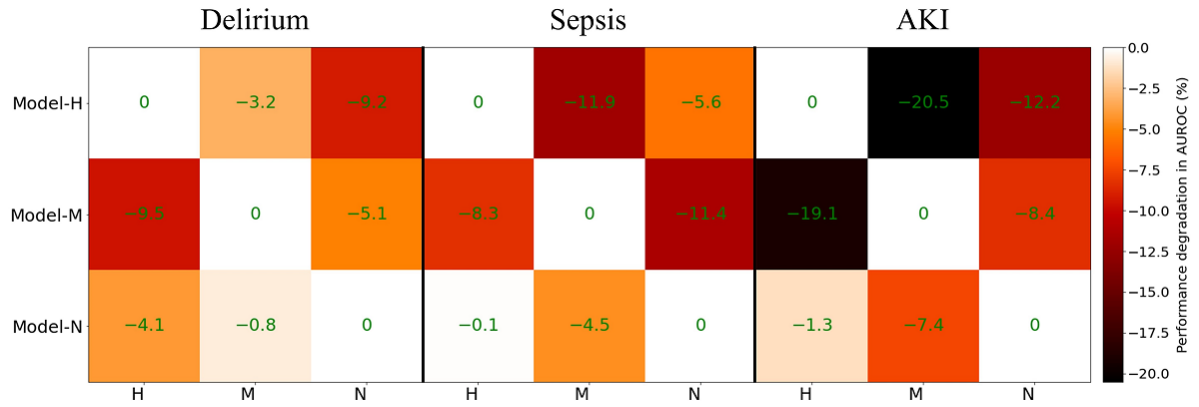
Performance degradation of a model trained in a certain hospital (rows) and deployed in another hospital (columns). The table was generated from the AUROC values from cross-hospital evaluation on the live data (Table S6 in Multimedia Appendix 1). AKI: acute kidney injury; AUROC: area under the receiver operating characteristic curve; H: Herz- und Diabeteszentrum Nordrhein-Westfalen Bad Oeynhausen; M: Marienhospital Stuttgart; N: Medius Klinik Nürtingen.

The detailed outcome of cross-hospital evaluation on prediction requests extracted from the live clinical workflow is provided in Table S7 in Multimedia Appendix 1. Prediction models for three different diseases (ie, delirium, sepsis, and AKI) were evaluated by comparing the AUROCs of different models at discharge. Figure 6 depicts the performance degradation of a model when trained in a certain hospital and deployed in another hospital. For each use case, AUROC values in a row are compared to the white cell in the same row, which indicates within-hospital performance. For example, when the delirium model trained on data from hospital H was deployed in hospital M (91.2%, column 2, row 1; Table S7 in Multimedia Appendix 1), the AUROC was 3.2 percentage points lower as compared to its performance in hospital H (94.4%, column 1, row 1; Table S7 in Multimedia Appendix 1). The largest performance degradation (–20.5 percentage points) was observed when the AKI model trained on data from hospital H was deployed in hospital M.

On average, the AUROC was 8 percentage points lower when a model was deployed in a hospital other than where it was trained (from 94.2% to 86.3%).

### Preliminary Evaluation of Clinical Soundness and Usefulness

The prediction models were installed in the live EHR systems of three different hospitals. These models generated predictions and triggered alerts in a live clinical workflow. Those alerts were displayed in the production EHR system and are currently under the evaluation of domain experts. A quantitative evaluation of the impact on clinical outcomes has not yet been performed. Nevertheless, the preliminary evaluation made by the domain experts assures the correctness and effectiveness of the predictions. A case study has been conducted to evaluate the performance of the delirium prediction models installed in hospital H [21]. Predictions made by the delirium risk prediction model following cardiac surgery were evaluated in the study. A cohort study investigating a larger population is also ongoing in the same hospital. The investigations identified that the prediction service could have an influence on anesthesia planning, as risk prediction is crucial for an early prevention strategy. The machine learning approach also improved postoperative care by enhanced screening efforts. In addition, the rest of this section presents our analysis of calibration and decision curves at hospital H, as well as our preliminary analysis on user feedback at hospital M.

### Calibration and Decision Curve Analysis

**Figure 7:**
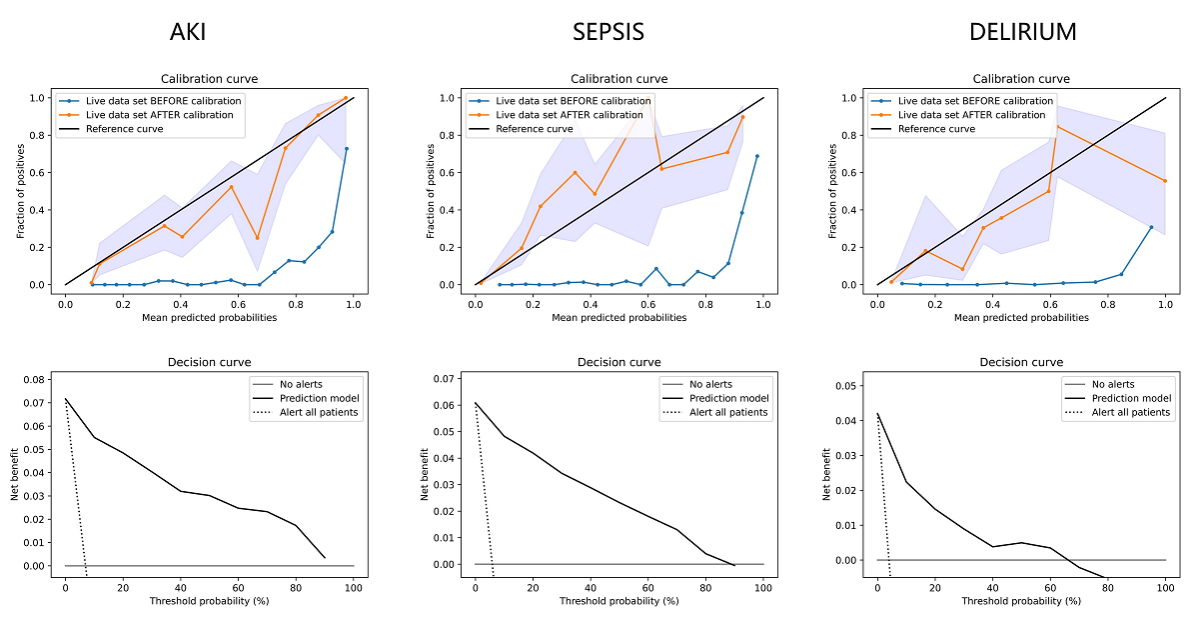
Calibration and decision curve analysis. The model and data were both from hospital H (Herz- und Diabeteszentrum Nordrhein-Westfalen Bad Oeynhausen). AKI: acute kidney injury.

Figure 7 shows the calibration and decision curve analysis for three use cases with the live data retrieved from hospital H. We first applied probability calibration [22,23] to generate calibration curves for each use case. The calibration curves plot the true frequency of the positive cases against its averaged predicted probability for each bin. We divided the probability into 10 bins. Predictions on the live data before and after probability calibration are shown. We used isotonic regression to perform the probability calibration. The calibration process used the first half of the live data, and the calibration curves and decision curves were generated using the second half of the live data. Due to the limited amount of available data, there are a few spikes in the calibrated curves. After the probability calibration, the decision curves [24,25] were generated to evaluate the net benefit of using the prediction models. The net benefits of the prediction models were compared with either “alert all patients” or “no alerts.” It can be observed that the prediction models were clinically useful when the threshold probability was below 90% for the AKI and sepsis use cases. For the delirium use case, the model had benefits when the threshold probability was below 70%.

**Figure 8:**
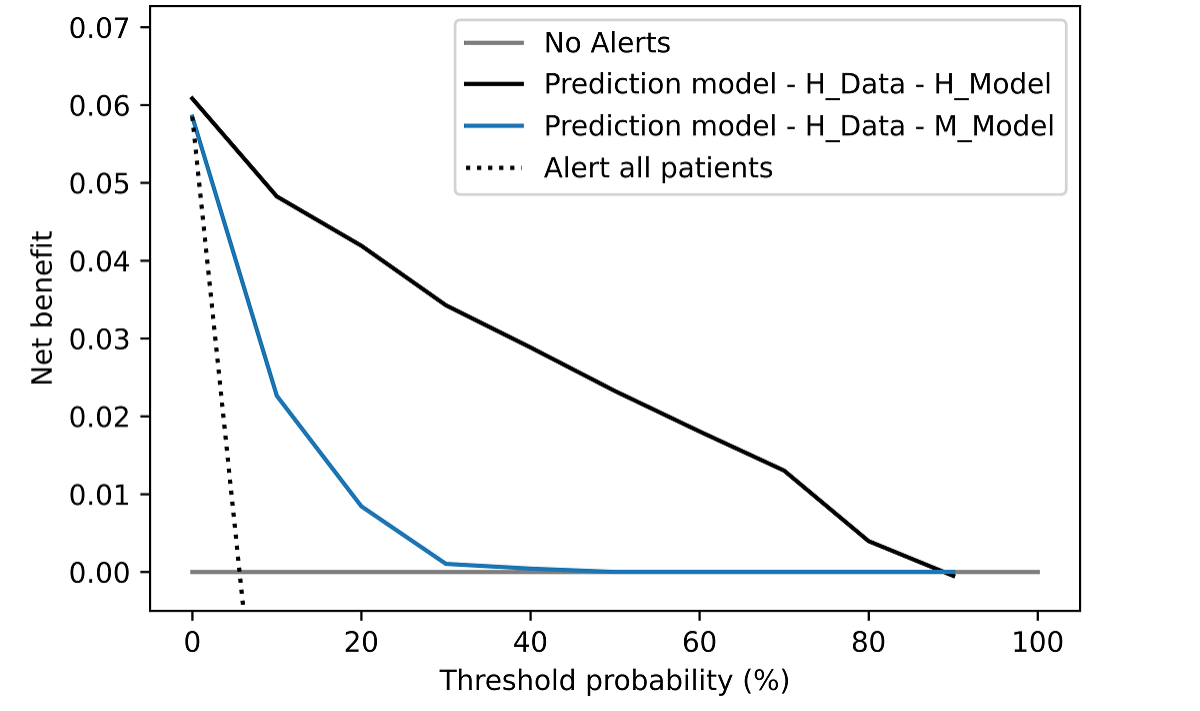
Decision curve analysis for the sepsis use case. Models trained at hospitals H and M, both using the live data from hospital H, are compared. H: Herz- und Diabeteszentrum Nordrhein-Westfalen Bad Oeynhausen; M: Marienhospital Stuttgart.

Figure 8 shows the decision curves for the prediction models trained at hospitals H and M on the sepsis use case. Both curves in Figure 8 were generated using the live data from hospital H, and the predicated probabilities were both calibrated. It can be observed that the model trained at hospital H was superior compared with the model trained at hospital M.

### Preliminary Analysis of User Feedback

When the prediction models were installed in the production EHR system, the end user was able to provide their feedback when they closed an alert. There were 134 feedback entries collected for the AKI use case at hospital M. More than one-third of the feedback entries (n=46, 34.3%) indicated that the users found the predictions useful. Details of the user feedback entries can be found in Figure S2 in Multimedia Appendix 1.

A total of 27.6% (37/134) of the alerts were considered to be false positives by the end users. This is a satisfactory result, considering the low incidence of AKI (838/8861, 9.46% at hospital M). Moreover, among 37 of those evaluated as false positive cases, 20 (54%) were already discharged and coded. Of these 20 discharged cases, 4 (20%) were actually coded as having AKI. This means that even if the physician disagrees with a prediction of high risk, there seems to still be a high risk that some patients will ultimately develop AKI, and our model can identify that risk.

In 38.1% (51/134) of the cases, the end users were already aware of the risk of AKI raised by the alert. There were two main reasons for this. Firstly, there was a clear gap between the time that the alert was created and the time that the feedback was given when the alert was closed. Secondly, alerts were only displayed in departments where the prediction service was activated; if a patient was transferred from a department where the prediction service was not activated, there would not be any alert displayed there.

## Discussion

### Principal Findings

The state of the art of machine learning development is to either design and train a single model and use it in different hospitals or design and train a specific model for a single hospital. We claim that defining a generic model design and training a specific instance of the model with data from a specific hospital has additional benefits for replicating the results. We observed performance degradation when a model was deployed in another hospital in our cross-hospital evaluation, a typical limitation of developing a single model for different hospitals. In the meantime, having a generic process and common model design to generate hospital-specific prediction models is a more robust solution. It resolves the intrinsic differences between different hospitals and guarantees sound performance at target hospitals. The evaluation of model performance in live clinical workflows assured the feasibility of such a generic approach, by checking the performance on three use cases at three different hospitals. In addition, by storing the logging data from live clinical workflows and having a common model design, the evaluation presented in this paper allows one to simulate the performance of a model in a live clinical workflow without it being installed on-site.

### Motivations

Machine learning–based prediction models are closely tied to the data used in the training process. This dependency largely restricts the reusability of a prediction model in other hospitals. A generic model that delivers unbiased performance in different hospitals is what machine learning scientists and clinicians earnestly long for but also often fail to achieve.

The prerequisite to generate a generic model that can be used in different hospitals is to achieve semantic interoperability that guarantees a common understanding between different EHR systems [26,27]. In order to achieve semantic interoperability, clinical terminologies need to be mapped onto a standard representation. However, a recent study [28] also showed safety risks related to the use of standard terminologies, such as LOINC (Logical Observation Identifiers Names and Codes), for interoperability between organizations due to inaccurate mappings.

In addition, a disease may have very different incidence rates in different hospitals due to the type and specialties of a hospital. Such a variety also results in different clinical workflows performed in different hospitals that determine the data the hospital records. A prediction model is, therefore, considered an algorithm that captures the knowledge and practice of the physicians of a hospital, by processing hospital-specific data that are presented in their specific representation. It is challenging to overcome the vulnerability of data shifts caused by diverse clinical workflows in different hospitals. Therefore, it is hard to maintain good performance when a model runs in a different hospital than the one within which it was trained, especially if the characteristics of the EHR data and the clinical workflow differ significantly. For example, the sepsis prediction of one particular vendor achieved satisfactory results in one hospital [29], but it was substantially worse when evaluated in another hospital [15].

We also observed performance degradation when a model was deployed in other hospitals in our cross-hospital evaluation. Therefore, instead of delivering a generic prediction model to different hospitals, we designed a generic procedure for prediction model development and applied it to different hospitals. Having a generic process to generate hospital-specific prediction models is a more robust solution; it resolves the intrinsic differences between different hospitals.

### Strengths

Evaluating prediction models in a live clinical workflow is crucial for validating their performances. To the best of our knowledge, we are the first to evaluate clinical prediction models on such a large scale in live clinical workflows. Such a thorough evaluation avoids overfitting to a certain disease or the settings of a particular hospital, thus allowing a fair, unbiased evaluation. The models deployed in the live clinical workflows delivered similar performances compared with those reported in our previous study [9], which were evaluated using retrospective data.

Sharing the same feature processing approach allows us to reuse the prediction requests by different prediction models. We, therefore, performed cross-hospital evaluation on three use cases in three different hospitals, mimicking the performance in a live clinical workflow rather than on retrospective data. To the best of our knowledge, this is the first study that performed cross-hospital evaluations on multiple use cases and simulated model performance in live clinical workflows.

### Limitations

This study had some limitations. First, our model development and evaluation on metrics reported in this paper lacked a dynamic evaluation to predict the risk within a time window of event onset. For example, the most widely used diagnostic criterion for AKI is based on changes in serum creatinine, as defined by the Kidney Disease: Improving Global Outcomes (KDIGO) guidelines [30]. Tomašev et al [31] reported an AKI prediction model that predicts the AKI risk 48 hours before the KDIGO-defined event. In the three use cases presented in this paper, delirium was considered a mental health disease that normally does not have a precise time of onset. We have developed an AKI prediction model based on retrospective data at our development site using KDIGO events as labels. The models were not deployed in the production system; however, their performance at two hospitals on retrospective data is enclosed (Table S8 in Multimedia Appendix 1). The AKI risk prediction curve of a sample patient during his medical stay is also provided (Figure S3 in Multimedia Appendix 1). For the sepsis prediction, we did not yet perform such a dynamic evaluation due to the lack of both scalable and accurate indicators of documented or suspected infection. Nevertheless, the AUROC for sepsis at the end of the day of admission ranged between 86.9% and 88.5% in the live system at the three different hospitals, which assures a satisfactory performance.

Second, although the metrics of the prediction models in the live clinical workflows were evaluated in different hospitals, the corresponding clinical outcomes in clinical practice are yet to be measured. Nevertheless, the preliminary clinical evaluation in hospital H affirms that there was a positive impact in the live clinical workflows, and a quantitative evaluation is scheduled as our next step of this work. The decision curve analysis and the preliminary analysis of user feedback also affirms the usefulness of our prediction models.

Third, machine learning approaches that are used to generate and validate prediction models are always data hungry [32]. Current external validation studies often suffer from small sample sizes compared with the large amount of predictor features [33]. The sample size presented in this paper was also relatively small compared with the number of predictor features used in our prediction model. Nevertheless, we also argue that for diseases with low incidence, it is difficult to obtain a large number of positive samples. The three use cases presented in this paper were running in live EHR systems for more than half a year, which we consider to be a reasonable amount of time. In addition, we ran evaluations on three different use cases at three different hospitals, which helps to justify the outcomes.

### Future Directions

Our future work will focus on evaluating the detailed clinical outcomes of prediction models in clinical practice. In addition, we will also evaluate the impact of different labeling strategies, such as defining AKI events with KDIGO criteria, in live systems.

### Conclusions

In this study, we found consistent performance of models when evaluated on retrospective and live data, and performance differences were observed in the cross-hospital evaluations. This ensures that designing a generic process for model development, implementing that design in a calibration tool, and generating hospital-specific prediction models with a common model design is a valid approach that guarantees model performance in different hospitals.

## Appendix

Multimedia Appendix 1 Supplementary materials.

## Abbreviations

AI: artificial intelligence
AKI: acute kidney injury
AUROC: area under the receiver operating characteristic curve
BERT: bidirectional encoder representations from transformers
CAM-ICU: Confusion Assessment Method for the Intensive Care Unit
EHR: electronic health record
FHIR: Fast Healthcare Interoperability Resources
GenoMed4All: Genomics and Personalized Medicine for All Though Artificial Intelligence in Haematological Diseases
hospital H: Herz- und Diabeteszentrum Nordrhein-Westfalen Bad Oeynhausen
hospital M: Marienhospital Stuttgart
hospital N: Medius Klinik Nürtingen
ICU: intensive care unit
KDIGO: Kidney Disease: Improving Global Outcomes
LOINC: Logical Observation Identifiers Names and Codes
NLP: natural language processing
PERSIST: Patients-Centered Survivorship Care Plan After Cancer Treatments Based on Big Data and Artificial Intelligence Technologies

## Data Availability

The patient data used in this evaluation from the three hospitals cannot be made publicly available due to patient protection. The code used to evaluate the model performance and to run the cross-hospital evaluation is available at GitHub [34].
