# Supplementary material for: Machine Learning–Based Prediction Models for Different Clinical Risks in Different Hospitals: Evaluation of Live Performance
Source: J Med Internet Res. 2022 Jun 7;24(6):e34295. doi: 10.2196/34295 (PMC9214618; doi:10.2196/34295)
Supplement: Multimedia Appendix 1 [file jmir_v24i6e34295_app1.docx]

Multimedia Appendix 1:

Evaluating live performance of machine learning based prediction models for different clinical risks: a study in live systems of different hospitals

Table of contents

[**Statistics of the logging information (evaluation samples)** 2](#_Toc100133734)

[**Characteristics of logging datasets and feature groups at the time of discharge** 3](#_Toc100133735)

[**Model acceptance criteria** 5](#_Toc100133736)

[**Sample log of a prediction request** 5](#_Toc100133737)

[**Response time for predictions** 6](#_Toc100133738)

[**Metrics of model performance evaluation (AUROC)** 7](#_Toc100133739)

[**Metrics of cross -hospital evaluation (AUROC)** 8](#_Toc100133740)

[**Preliminary user feedback** 9](#_Toc100133741)

[**KDIGO evaluation for AKI predictions** 10](#_Toc100133742)

[**References:** 11](#_Toc100133743)

# **Statistics of the logging information (evaluation samples)**

Table S1. Statistics of the logging information (evaluation samples)

|  | Delirium | | Sepsis | | AKI | |
| --- | --- | --- | --- | --- | --- | --- |
|  | Log period | Incidence:  cases /  samples | Log period | Incidence:  cases /  samples | Log period | Incidence:  cases /  samples |
| Hospital N | 2021-02-11- 2021-08-06 | 97/  4418 | 2021-02-17 - 2021-08-06 | 70/  4107 | 2021-02-11 - 2021-08-06 | 335/  4412 |
| Hospital M | 2021-01-29 - 2021-08-11 | 268/  8866 | 2021-01-29 - 2021-08-11 | 120/  8866 | 2021-01-29 - 2021-08-11 | 838/  8861 |
| Hospital H | 2021-01-21 - 2021-08-06 | 141/  4660 | 2021-02-01 - 2021-08-06 | 198/  4605 | 2021-01-21 - 2021-08-06 | 259/  4646 |

Table S1 provides the statistics of the log period of each prediction service, as well as the incidence for closed patient stays. The number of samples correspond to the number of in-hospital patient stays. We use diagnosis codes at discharge as labels for this evaluation. Since there could be a delay in assigning the diagnosis codes to discharged patients, we only include patients that are discharged more than 2 months before the model performance evaluation in the live EHR system.

# **Characteristics of logging datasets and feature groups at the time of discharge**

Table S2 shows the characteristics of the feature groups in the retrospective training data. Table S3 shows the characteristics of the feature groups, based on an analysis of observations contained in the log files, at the time of discharge. It represents the characteristics of the data from the live clinical workflow. The characteristics of the observations in the retrospective training data are consistent with the data logged from the live clinical workflow.

Table S2. Characteristics of feature groups in retrospective training datasets

| Use case | Delirium | | | Sepsis | | | AKI | | |
| --- | --- | --- | --- | --- | --- | --- | --- | --- | --- |
| Hospital name | H | M | N | H | M | N | H | M | N |
| Number of records | 6456 | 19230 | 46218 | 13764 | 18600 | 37704 | 33198 | 62766 | 116670 |
| Age, median | 66.0 | 61.3 | 64.8 | 64.8 | 60.1 | 63.8 | 65.1 | 60.3 | 64.0 |
| Female sex, no, (%) | 2062  (32%) | 10492  (55%) | 24243  (53%) | 4424  (32%) | 10343  (56%) | 19663  (52%) | 11064  (33%) | 35246  (56%) | 61393  (53%) |
| Normal admission | 5768  (89%) | 9900  (52%) | 21147  (46%) | 12233  (89%) | 9635  (52%) | 17087  (45%) | 29539  (89%) | 34221  (55%) | 55359  (47%) |
| Emergency admission | 611  (10%) | 9249  (48%) | 24816  (54%) | 1387  (10%) | 8873  (48%) | 20418  (54%) | 3288  (10%) | 28239  (45%) | 60662  (52%) |
| History of diagnosis, no, (%) | 2556  (40%) | 8819  (46%) | 26435  (57%) | 5527  (40%) | 8372  (45%) | 21429  (57%) | 13366  (40%) | 28152  (45%) | 65013  (56%) |
| Medication, no, (%) | 1137  (18%) | 0  (0%) | 2617  (6%) | 1919  (14%) | 0  (0%) | 2686  (7%) | 4360  (13%) | 0  (0%) | 5834  (5%) |
| Lab Results, no, (%) | 6305  (98%) | 17449  (91%) | 44084  (95%) | 13472  (98%) | 16873  (91%) | 35985  (95%) | 32473  (98%) | 56312  (90%) | 111049  (95%) |
| Vital Sign, no, (%) | 3699  (57%) | 13753  (72%) | 18130  (39%) | 7201  (52%) | 13168  (71%) | 14911  (40%) | 17262  (52%) | 44541  (71%) | 45507  (39%) |
| Named Clinical Entities, no, (%) | 6277  (97%) | 18966  (99%) | 36018  (78%) | 13367  (97%) | 18364  (99%) | 29381  (78%) | 27879  (84%) | 61938  (99%) | 90470  (78%) |

* Each training dataset has a 1:1 ratio of records with and without the targeted disease.

Table S3. Characteristics of feature groups from the live data set (evaluation samples)

| Use case | Delirium | | | Sepsis | | | AKI | | |
| --- | --- | --- | --- | --- | --- | --- | --- | --- | --- |
| Hospital name | H | M | N | H | M | N | H | M | N |
| Number of records | 4660 | 8866 | 4418 | 4605 | 8866 | 4107 | 4646 | 8861 | 4412 |
| Age group, mean | 5.6 | 5.1 | 5.1 | 5.6 | 5.1 | 5.1 | 5.6 | 5.1 | 5.1 |
| Female sex, no, (%) | 1545 (33%) | 4925 (56%) | 2559 (58%) | 1524 (33%) | 4925 (56%) | 2362 (58%) | 1542 (33%) | 4921 (56%) | 2554 (58%) |
| Normal Admission | 4122 (88%) | 4582 (52%) | 2196 (50%) | 4067 (88%) | 4582 (52%) | 2032 (49%) | 4109 (88%) | 4580 (52%) | 2196 (50%) |
| Emergency Admission | 538 (12%) | 4284 (48%) | 2222 (50%) | 538 (12%) | 4284 (48%) | 2075 (51%) | 537 (12%) | 4281 (48%) | 2216 (50%) |
| History of diagnosis, no, (%) | 2469 (53%) | 6434 (73%) | 3917 (89%) | 2445 (53%) | 6437 (73%) | 3643 (89%) | 2485 (53%) | 6487 (73%) | 3922 (89%) |
| Medication, no, (%) | 1519 (33%) | 0 (0%) | 0 (0%) | 1585 (34%) | 0 (0%) | 0 (0%) | 1708 (37%) | 0 (0%) | 0 (0%) |
| Lab results, no, (%) | 4521 (97%) | 7980 (90%) | 4199 (95%) | 4471 (97%) | 7980 (90%) | 3890 (95%) | 4505 (97%) | 7959 (90%) | 4172 (95%) |
| Vital signs, no, (%) | 3733 (80%) | 6051 (68%) | 3111 (70%) | 3694 (80%) | 6051 (68%) | 2904 (71%) | 3716 (80%) | 6048 (68%) | 3114 (71%) |
| Named Clinical Entities, no, (%) | 4581 (98%) | 8551 (96%) | 3969 (90%) | 4529 (98%) | 8552 (96%) | 3687 (90%) | 4568 (98%) | 8555 (97%) | 3962 (90%) |

# **Model acceptance criteria**

Table S4. Model acceptance criteria

| Use case | Criteria to define threshold | Acceptance criteria |
| --- | --- | --- |
| Delirium | sensitivity = 80% (±5%) | specificity > 85% |
| Sepsis | precision = 25% (±5%) | sensitivity > 50% |
| AKI | precision = 25% (±5%) | sensitivity > 60% |

Table S4 shows the acceptance criteria for the three use cases. Firstly, a threshold that identifies positive or negative predictions is defined based on the given criteria. For example, for the delirium use case, the threshold is calculated in order to guarantee that sensitivity is around 80%. Based on the generated threshold, the acceptance criterium is that the specificity must be greater than 85%.

The acceptance criteria are evaluated on department level. Departments that meet the acceptance criteria can trigger the prediction service, so that an alert will be displayed when the predicted probability exceeds the threshold. For those department that fail to meet the acceptance criteria, the prediction service still produces risk predictions, however, those predictions will not trigger an alert even it exceeds the threshold.

# **Sample log of a prediction request**

Table S5 shows an excerpt of logging information of a prediction request. The date and time of prediction is stored in the log entry heading, followed by the ID of the corresponding medical case (1234567, anonymized in this excerpt). The observation sent to the prediction model (starting with ‘GENDER-MALE’), is kept intact in the logging file.

Table S5. Excerpt of a prediction request log

| '{"log":"[2021-07-17 09:36:32,568] [CASE 1234567] [INFO] [/t2t/server.py:83] REQUEST 4784726:  GENDER-MALE AGE_GROUP-7 DEPARTMENT-1900 ADMISSION_KIND-1  ICD_HISTORICAL-K80.00 ICD_HISTORICAL-K21.9 ICD_HISTORICAL-E13.91  ICD_HISTORICAL-D86.0 ICD_HISTORICAL-R55 ICD_HISTORICAL-I25.9  LAB_RESULT-BZ_POCT_15_15-N LAB_RESULT-BZ_POCT_07_00-H LAB_RESULT-BZ_POCT_16_30-N  LAB_RESULT-BZ_POCT_11_30-N LAB_RESULT-BZ_POCT_07_45-N LAB_RESULT-BZ_POCT_17_00-N  SELECTED_KEYWORD-DIABETES SELECTED_KEYWORD-TUMOR SELECTED_KEYWORD-CARCINOMA  DISORDER-KARZINOM DISORDER-PLATTENEPITHELKARZINOM DISORDER-TV VITAL_SIGN-TEMPERATURE-L-SOMETIMES  VITAL_SIGN-DIASTOLIC_BLOOD_PRESSURE-N-SOMETIMES VITAL_SIGN-DIASTOLIC_BLOOD_PRESSURE-H-SOMETIMES\\n",  "stream":"stderr","time":"2021-07-17T09:36:32.568849435Z"}\n', |
| --- |

# **Response time for predictions**

The response time for predictions is evaluated in two production systems that only use CPUs (no GPUs). More than 95% of the prediction requests can be processed within one second.


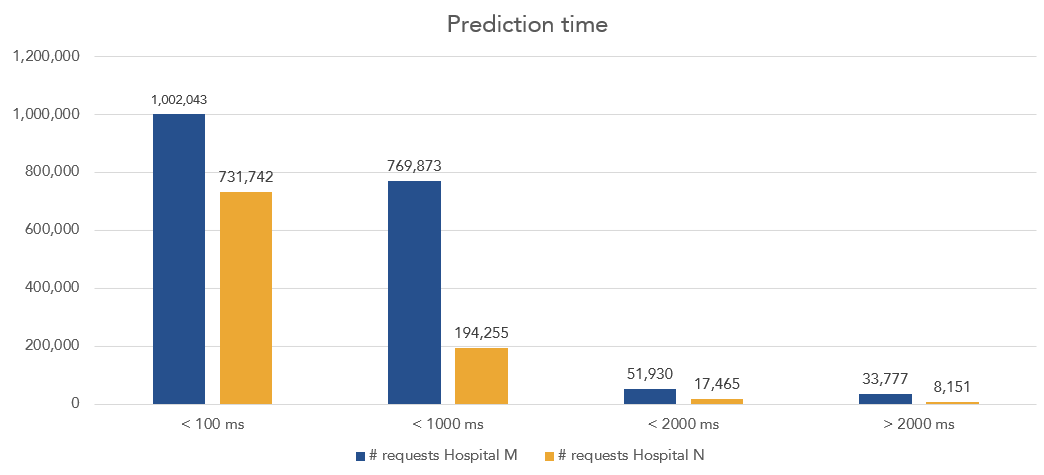


Figure S1. Response time for predictions

# **Metrics of model performance evaluation (AUROC)**

Table S6 compares the AUROC of the three different prediction models on the retrospective data with their performance in live clinical workflows. The AUROC is generated in three hospitals at the end of the day of admission and at discharge. The AUROC is lower at the end of the day of admission, largely due to the limited data available for predictions at the start of the hospital stay. In general, there is no big difference between the AUROC achieved with retrospective data and with data from the live clinical workflow. Detailed analysis of the difference in performance is summarized in Figure 4 of the main manuscript.

Table S6. Evaluation of model performance

|  |  | **DELIRIUM** | | **SEPSIS** | | **AKI** | |
| --- | --- | --- | --- | --- | --- | --- | --- |
|  |  | **admission AUROC** | **discharge AUROC** | **admission AUROC** | **discharge AUROC** | **admission AUROC** | **discharge AUROC** |
| **N** | **Live- analysis** | 80.9% [79.7- 82.1] | 93.4% [92.7- 94.2] | 88.5% [87.5- 89.5] | 94.5% [93.8- 95.2] | 86.8% [85.8- 87.9] | 91.5% [90.7- 92.3] |
|  | **Retro- analysis** | 85.26% [85.0–85.5] | 93.09% [92.9–93.2] | 89.32% [89.1–89.5] | 95.55% [95.4–95.7] | 83.65% [83.4–83.9] | 88.09% [87.9–88.3] |
| **M** | **Live- analysis** | 83.5% [82.7- 84.3] | 94.6% [94.2- 95.1] | 87.5% [86.8- 88.2] | 96.6% [96.2- 97.0] | 83.2% [82.4- 84.0] | 89.5% [88.9- 90.1] |
|  | **Retro- analysis** | 84.57% [84.3–84.8] | 96.74% [96.6–96.9] | 87.14% [86.9–87.4] | 96.48% [96.3–96.6] | 87.20% [87.0–87.4] | 93.41% [93.2–93.6] |
| **H** | **Live- analysis** | 69.9% [68.6- 71.3] | 94.4% [93.8- 95.1] | 86.9% [85.9- 88.0] | 97.5% [97.1- 98.0] | 80.3% [79.2- 81.5] | 95.1% [94.5- 95.7] |
|  | **Retro- analysis** | 75.20% [74.7–75.7] | 98.03% [97.9–98.2] | 82.67% [82.3–83.1] | 96.17% [96.0–96.4] | 80.37% [79.9–80.8] | 95.75% [95.5–96.0] |

# **Metrics of cross -hospital evaluation (AUROC)**

Table S7. Cross-hospital evaluation (AUROC (%))

|  | **Delirium** | | | **Sepsis** | | | **AKI** | | |
| --- | --- | --- | --- | --- | --- | --- | --- | --- | --- |
|  | **H**  ***(141/ 4660)*** | **M**  ***(268/ 8866)*** | **N**  ***(97/ 4418)*** | **H**  ***(198/ 4605)*** | **M**  ***(120/ 8866)*** | **N**  ***(70/ 4107)*** | **H**  ***(259/ 4646)*** | **M**  ***(838/ 8861)*** | **N**  ***(335/ 4412)*** |
| **Model H** | **94.4 [93.8, 95.1]** | 91.2 [90.6, 91.8] | 85.2 [84.2, 86.3] | **97.5 [97.1, 98]** | 85.6 [84.8, 86.3] | 91.9 [91.1, 92.8] | **95.6 [95, 96.2]** | 75.1 [74.2, 76.0] | 83.4 [82.3, 84.5] |
| **Model M** | 85.1 [84.0, 86.1] | **94.6 [94.2, 95.1]** | 89.5 [88.6, 90.4] | 88.3 [87.3, 89.2] | **96.6 [96.2, 97.0]** | 85.2 [84.1, 86.3] | 70.4 [69.1, 71.7] | **89.5 [88.9, 90.1]** | 81.1 [79.9, 82.2] |
| **Model N** | 89.3 [88.4, 90.2] | 92.6 [92.1, 93.2 | **93.4 [92.7, 94.2]** | 94.4 [93.7, 95] | 90.0 [89.4, 90.6] | **94.5 [93.8, 95.2]** | 90.2 [89.3, 91.1] | 84.1 [83.4, 84.9] | **91.5 [90.7, 92.3]** |

Table S7 summarizes the outcome of cross-hospital evaluation on prediction requests extracted from the live clinical workflow. Rows represent a prediction model for delirium, sepsis or AKI, trained in the indicated hospital. Columns represent the hospitals where the model was evaluated. Each cell contains the AUROC and the 95% confidence interval. Numbers in bold indicate within-hospital performance. The incidence (number of cases / number of samples) referring to the evaluation dataset are also indicated.

# **Preliminary user feedback**


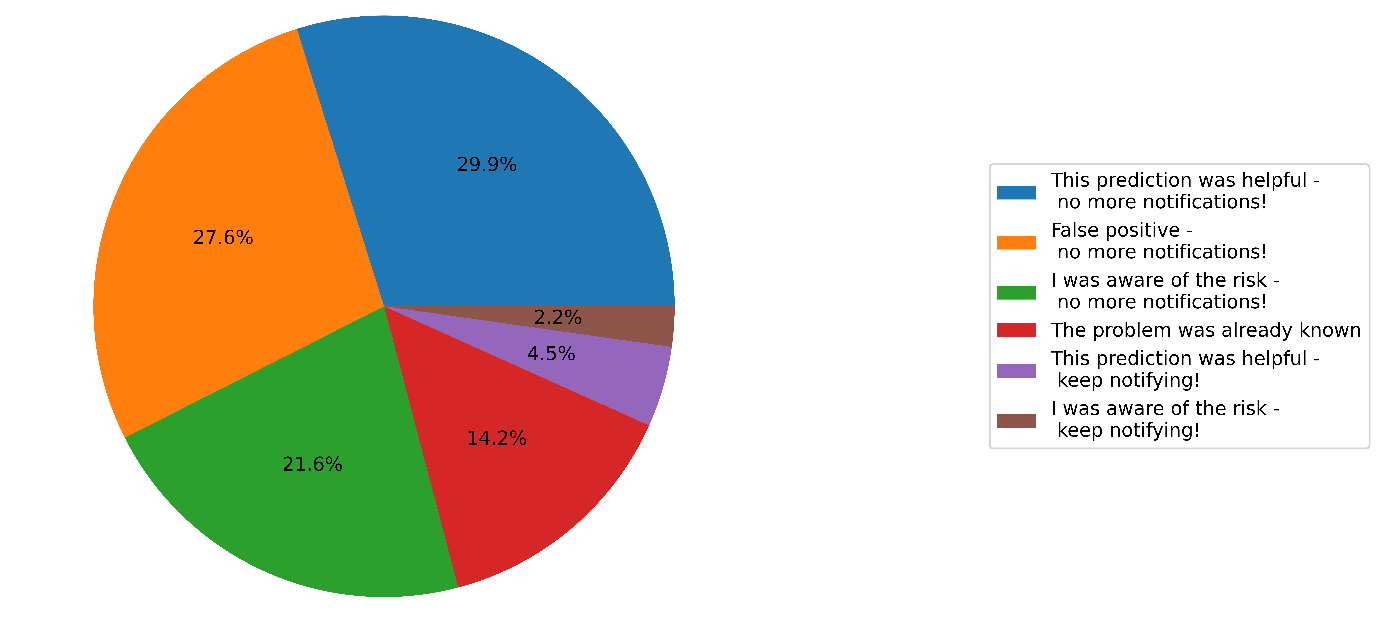


Figure S2. Preliminary user feedback on AKI prediction model at hospital M

When the prediction models are installed in the production EHR system, alerts will be triggered when the predicted probability exceeds the predefined threshold. The end-user can provide their feedback when they close an alert, to indicate the usefulness of the prediction as well as whether the system should keep notifying them of the risk. Figure S2 shows the user feedback on AKI prediction model at hospital M. There are 134 feedbacks collected on the AKI use case. More than one-third of the feedbacks (blue and violet) found the predictions useful, most of them (blue) do not need further notification, a small fraction (violet) want to keep notifying.

# **KDIGO evaluation for AKI predictions**

Figure S3 shows the AKI predictions before and after a KDIGO-defined event.

- The upper chart shows the changes of the creatinine value during a medical stay. The yellow curve indicates an AKI event calculated based on the KDIGO criteria.
- The lower chart shows the scores of the predictions made with the AKI prediction model. The red line indicates the threshold to trigger an alert, the yellow curve indicates an AKI event predicted by the prediction model.

It can be observed that the model successfully predicted the AKI event one day before the calculated KDIGO event, and continued positive predictions after the event.


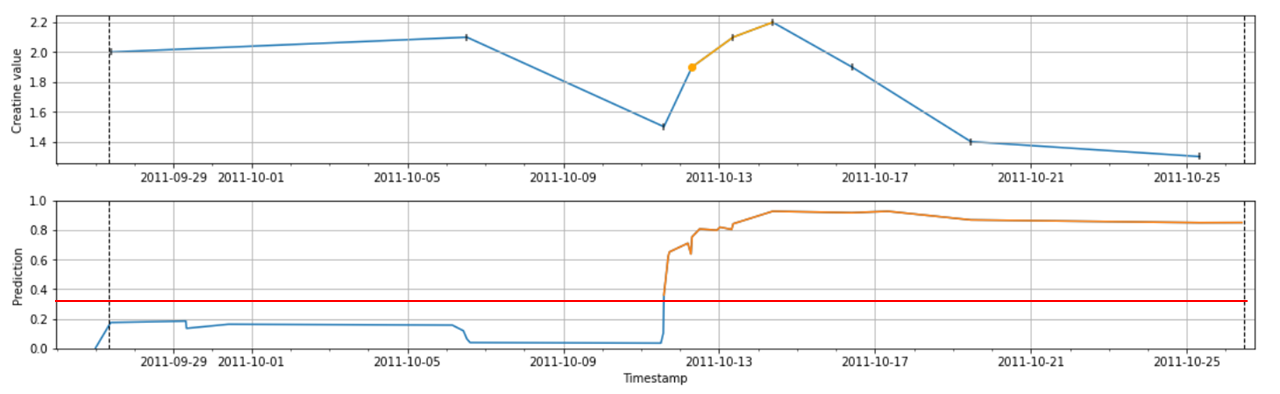


Figure S3. AKI predictions before and after a KDIGO-defined event

Table S8 shows the model performance 6 hours before the AKI event that is defined by the KDIGO criteria and 24 hours after admission. Each row corresponds to the prediction of one or several KDIGO-defined event groups, as well as the prediction period. The cumulative result indicates per medical case, if a prediction made during the specified period is positive, then the entire medical case is considered as a positive prediction. The KDIGO AKI models deliver generally satisfactory results compared with most of the previous reports [2-5] with an AUROC ranging from below 80% to 89% before the AKI event. Tomašev et al [1] reported the best performance on AUROC in predicting KDIGO based AKI events: for KDIGO stage 2 or 3 with AUROC 97.1% 24 hours in advance. The limitation of their work is that only patients with extensive health records before admission are included, which is not always the case in the live clinical workflow [1, 5]. Our model provides a comparable performance in hospital H for KDIGO stage 2 or 3 with AUROC 96.48% 6 hours in advance. Moreover, although the model generation process is developed on the development site, the automatically generated model in hospital H delivers better performance with an AUROC of 4 percentage points higher on average.

Table S8. Model performance before KDIGO onset

| KDIGO Stage | Prediction period | AUROC (CI 95%)  (development site) | AUROC (CI 95%)  (hospital H) |
| --- | --- | --- | --- |
| 1-2-3 | >= 6hr before onset | 87.01  [86.66-87.35] | 93.30  [93.01-93.59] |
| 1-2-3 | >= 6hr before onset (cumulative) | 83.40  [83.02-83.78] | 88.39  [88.01-88.77] |
| 1-2-3 | <= 24hr after admission | 90.20  [89.90-90.49] | 92.33  [92.02-92.65] |
| 1-2-3 | <= 24hr after admission (cumulative) | 84.56  [84.19-84.92] | 85.66  [85.27-86.06] |
| 2-3 | >= 6hr before onset | 91.50  [91.21-91.79] | 96.48  [96.25-96.71] |
| 2-3 | >= 6hr before onset (cumulative) | 87.33  [86.98-87.67] | 92.99  [92.68-93.31] |
| 3 | >= 6hr before onset | 92.90  [92.64-93.17] | 97.00  [96.79-97.21] |
| 3 | >= 6hr before onset (cumulative) | 88.59  [88.26-88.92] | 93.97  [93.67-94.26 |

# **References:**

[1] Tomašev, N., Glorot, X., Rae, J. W., Zielinski, M., Askham, H., Saraiva, A., ... & Mohamed, S. (2019). A clinically applicable approach to continuous prediction of future acute kidney injury. Nature, 572(7767), 116-119.

[2] Hodgson, L. E., Sarnowski, A., Roderick, P. J., Dimitrov, B. D., Venn, R. M., & Forni, L. G. (2017). Systematic review of prognostic prediction models for acute kidney injury (AKI) in general hospital populations. BMJ open, 7(9), e016591.

[3] Kate, R. J., Pearce, N., Mazumdar, D., & Nilakantan, V. (2020). A continual prediction model for inpatient acute kidney injury. Computers in biology and medicine, 116, 103580.

[4] Mohamadlou, H., Lynn-Palevsky, A., Barton, C., Chettipally, U., Shieh, L., Calvert, J., ... & Das, R. (2018). Prediction of acute kidney injury with a machine learning algorithm using electronic health record data. Canadian journal of kidney health and disease, 5, 2054358118776326.

[5] Rank, N., Pfahringer, B., Kempfert, J., Stamm, C., Kühne, T., Schoenrath, F., ... & Meyer, A. (2020). Deep-learning-based real-time prediction of acute kidney injury outperforms human predictive performance. NPJ digital medicine, 3(1), 1-12.
